# Longitudinal Sleep Patterns and Risk of Type 2 Diabetes Mellitus

**DOI:** 10.1002/dmrr.70237

**Published:** 2026-10-12

**Authors:** Ying Zhang, Zhen'ge Chang, Shuang Zhang, Yingying Guo, Qian Guo, Feiyu Zhang, Yating Chen, Fang Luo, Kefei Dou, Caixia Guo

**Affiliations:** ^1^ Cardiovascular Center Beijing Tongren Hospital Capital Medical University Beijing China; ^2^ Cardiometabolic Medicine Center Fuwai Hospital National Center for Cardiovascular Diseases, Chinese Academy of Medical Sciences & Peking Union Medical College Beijing China; ^3^ Center for Coronary Artery Disease, Division of Cardiology Beijing Anzhen Hospital Capital Medical University Beijing China; ^4^ State Key Laboratory of Cardiovascular Disease Beijing China

**Keywords:** public health, repeated assessment, sleep pattern transition, type 2 diabetes mellitus

## Abstract

**Aims:**

Evidence relating sleep characteristics to type 2 diabetes mellitus (T2DM) has largely been based on single assessments. We examined whether transitions in a multidimensional healthy sleep score between two assessments were associated with incident T2DM and explored the relationship of the triglyceride‐glucose (TyG) index to this association.

**Materials and Methods:**

We conducted a landmark analysis among 60,249 UK Biobank participants with sleep assessed at visits 1 and 2. Visit 2 was defined as time zero for follow‐up. Sleep pattern transitions were classified according to poor, intermediate, or healthy sleep status at each assessment. Cox proportional hazards models were used to estimate associations with incident T2DM. TyG analysis was treated as exploratory.

**Results:**

During a median follow‐up of 4.2 years, 1284 incident T2DM cases occurred. Compared with stable healthy sleep, stable poor sleep was associated with higher risk (hazard ratio [HR], 2.07; 95% confidence interval [CI], 1.32–3.27). Baseline‐stratified analyses did not show higher risk for improvement from poor or intermediate sleep relative to remaining in the same baseline category. The full‐cohort spline showed evidence of nonlinearity (*p* for nonlinearity < 0.001), and TyG analyses were exploratory.

**Conclusions:**

Sleep pattern transitions between the two assessments were associated with incident type 2 diabetes, and the association differed by baseline sleep status. Repeated sleep assessment may complement single‐time‐point evaluation in understanding the role of sleep in diabetes risk.

## 1 Introduction

Sleep is fundamental to physiological homoeostasis, yet sleep quality has become a major public health challenge, with global prevalence of disturbances ranging from 8.3% to 45% [1]. Recent research using a multidimensional framework encompassing duration, chronotype, and clinical symptoms characterised over 40% of the UK population as poor sleepers [2]. A substantial body of evidence links these prevalent sleep issues to adverse health outcomes, including an elevated risk of cardiometabolic conditions, cardiovascular disease (CVD), and mortality [3, 4]. In light of this evidence and after evaluating multiple metrics of sleep measurement, the American Heart Association (AHA) added sleep duration as an eighth metric of cardiometabolic health, elevating AHA's Life's Simple 7 to Life's Essential 8 in 2022 [5].

Diabetes mellitus (DM) stands as a predominant non‐communicable disease and a formidable public health challenge [6]. Over the last 40 years, its global prevalence has nearly quadrupled, currently affecting an estimated 537 million adults (11.1% worldwide), with further increases projected [7]. Unhealthy lifestyles encompass poor diet, high stress, and sedentary behaviour [8]; suboptimal sleep habits are also increasingly recognized [9]. However, existing research has largely focused on static sleep dimensions, overlooking changes across repeated assessments. Given that sleep evolves with ageing, lifestyle, and health status, recent evidence suggests that repeated sleep assessment may provide information beyond a single measurement when evaluating cardiometabolic health [10]. Therefore, examining transitions between repeated sleep assessments may improve understanding of associations between sleep characteristics and diabetes risk.

To address these gaps, this study used repeated UK Biobank sleep assessments to: (1) evaluate associations between sleep pattern transitions and incident T2DM with attention to baseline sleep status; (2) examine signed healthy sleep score (HSS) change as a secondary exposure; and (3) explore the relationship of the TyG index to the observed association.

## 2 Materials and Methods

Study population: Participants were sourced from the UK Biobank (UKB), which recruited more than 500,000 adults aged 37–73 years across England, Scotland, and Wales from 2006. Details of the design have been reported previously [11]. The analytic sample comprised 60,249 participants with sleep assessed at both visits; cohort selection is summarised in Supporting Information S1: Figure S1, and study fields are listed in Supporting Information S1: Table S1. This study used a landmark design in which sleep pattern transitions were defined between visits 1 and 2, and visit 2 was time zero for subsequent T2DM follow‐up. Participants were required to be alive and free of diabetes at visit 2 to enter the landmark risk set. The interval between assessments was a median of 3650 days (interquartile range [IQR], 3030–4416; range, 1400–6175).

Assessment of sleep metrics: The healthy sleep pattern was constructed from five aspects of sleep behaviours derived from the UK Biobank sleep touchscreen questionnaires. At both visits, one point was assigned for each low‐risk response: morning or more‐morning‐than‐evening chronotype (Field 1180), 7–8 h/day sleep duration (Field 1160), never/rarely or sometimes insomnia (Field 1200), no snoring (Field 1210), and never/rarely or sometimes daytime dozing (Field 1220). The HSS was calculated by summing the five sleep factors, a method previously used in other cohort studies [2]. The HSS ranged from 0 to 5, with a higher score indicating a healthier sleep pattern. According to the previous study, we defined three groups of sleep patterns, including poor, intermediate, and healthy sleep patterns, corresponding to the HSS of 0–1, 2–3, and 4–5, respectively.

Assessment of changes in sleep metrics: The exposures were the change in sleep patterns and the HSS derived from two time points, visit 1 (Instance 0, 2006–2010) and visit 2 (Instance 2, 2014+). In terms of the HSS, we calculated the signed difference as visit 2 HSS minus visit 1 HSS and then divided the participants into no‐change, increase, and decrease groups. Transition categories served as the primary exposure; signed HSS change categories were examined as a secondary exposure. For the change in sleep patterns, we defined five categories of sleep pattern changes: stable poor, worsening, stable intermediate, optimising, and stable healthy sleep patterns. The detailed categories are shown in Supporting Information S1: Table S2.

Assessment of Type 2 diabetes mellitus: Incident T2DM after the visit‐2 landmark was defined using the three‐character International Classification of Diseases, Tenth Revision (ICD‐10) codes, based on linkage data of the primary care records, hospital inpatient records, death records, and self‐reported diagnoses [12]. The outcome dates of T2DM diagnoses/codes were defined as the first recorded across all the above data sources, generated by a standard algorithm from the UKB team. Follow‐up for individuals extended from visit 2 (time zero) until the first of: T2DM diagnosis, loss to follow‐up, death, or the end of the study period (March 31, 2023). Diabetes present at or before the visit‐2 landmark was excluded. The details are provided in Supporting Information S1: Table S1 and Figure S1.

Covariates: Baseline variables included sex recorded as male or female, race or ethnicity recorded as white or not white, age at baseline, and body mass index (BMI). Obesity was defined as BMI ≥ 30 according to WHO categories. Socioeconomic status was assessed using the Townsend deprivation index (TDI) based on national census output for each postal code area. Individual‐level socioeconomic status was classified by the average annual household income before tax in pounds sterling with three categories: < £18,000, £18,000–£51,999, and > £52,000. Self‐reported lifestyle factors included smoking status (never, current, or previous), alcohol consumption (abstainer, light or moderate, and heavy or abusive), and physical activity (assessed by the Minnesota Leisure Time Activity Questionnaire and classified into four levels: never, low activity, medium activity, and high activity [13]). Alcohol intake was calculated from the average monthly or weekly number of alcoholic drinks, and participants were then classified using physician‐derived definitions into three groups: abstainer (0 drinks/week), light or moderate drinker (0–15.4 drinks/week), and heavy or abusive (> 15.4 drinks/week) drinker. Comprehensive details are provided in previous studies [14]. History of hypertension and hyperlipidaemia was ascertained from self‐reported diagnoses and ICD‐10 codes from linked hospitalisation records. The polygenic risk score (PRS) for T2DM from the UKB database has been previously described [15]. Laboratory measures included glucose and triglyceride (both mmol/L). The analytical platforms for laboratory tests included Beckman Coulter AU5800 (for glucose and triglyceride). We further calculated the triglyceride‐glucose (TyG) index to serve as a surrogate marker of insulin resistance [16]. The TyG index was calculated as ln[triglyceride (mg/dL) × glucose (mg/dL)/2]. All covariates were assessed at visit 1.

Statistical analyses: Baseline characteristics were presented as means ± standard deviation or median (IQR) for continuous variables and as frequencies (percentages) for categorical variables. Between‐group comparisons used analysis of variance for continuous variables and chi‐square tests for categorical variables. For variables with less than 5% missingness, missing covariates were imputed in 20 datasets by fully conditional specification, using logistic or multinomial regression for categorical variables and predictive mean matching for continuous variables; estimates were pooled using Rubin's rules. Variables with 5% or greater missingness were categorised as ‘unknown’. Missing data for all covariates were < 5%, except for university degree (7.8%), household income (9.7%), and alcohol consumption status (16.9%).

We employed Cox proportional hazards models to estimate hazard ratios (HRs) and 95% CIs. The proportional hazards assumption was assessed using Schoenfeld residuals. First, we conducted separate Cox regression analyses to examine the associations of sleep patterns at each of the two visits with the risk of diabetes. Subsequently, using participants with stable healthy sleep patterns as the reference group, we investigated the association between sleep‐pattern transitions and the risk of incident diabetes. To assess the robustness of the findings across populations with different baseline sleep quality, we performed analyses using two grouping strategies: baseline healthy versus non‐healthy sleep, and baseline poor, intermediate, versus healthy sleep. Three models were constructed with progressive adjustment for potential confounders. Model 1 was adjusted for age, sex, and ethnicity. Model 2 was additionally adjusted for household income, TDI, education level, employment status, smoking status, alcohol consumption, physical activity, and obesity. Model 3 was further adjusted for history of hypertension, history of hyperlipidaemia, and the PRS for T2DM. A directed acyclic graph (DAG) guided the adjustment set by identifying demographic, socioeconomic, lifestyle, clinical, and genetic factors as potential common causes of sleep characteristics and T2DM (Supporting Information S1: Figure S2).

We also evaluated the association between the changes in the HSS and diabetes risk, examined this association within subgroups defined by baseline sleep quality, and explored potential nonlinear relationships using restricted cubic splines. For the full‐cohort spline, three knots were placed at signed HSS changes of −1, 0, and 1, with 0 as the reference; overall and nonlinear associations were evaluated using likelihood‐ratio tests. Furthermore, we analysed the associations between changes in the five individual components of the HSS and diabetes risk. To investigate the potential role of insulin resistance, we examined associations between sleep‐pattern transitions and the visit‐1 TyG index. Because TyG was measured at visit 1, before the sleep transition, the TyG analyses are exploratory.

Stratified analyses were conducted to examine potential effect modification by sex (male/female), age (< 65/≥ 65 years), race or ethnicity (White/non‐White), BMI (< 25/25–29.9/≥ 30 kg/m^2^), current smoking status (yes/no), current alcohol consumption (no/yes/unknown), hypertension (yes/no), dyslipidemia (yes/no), and the PRS for T2DM (high/intermediate/low). For subgroup analyses, the five‐level sleep‐transition code was entered as a numeric term; HRs represent a one‐category increase. Interaction *p* values were pooled across imputations and corrected across nine tests using the Benjamini–Hochberg method.

Sensitivity analyses included complete‐case analysis, alternative BMI specifications using continuous linear and nonlinear terms, exclusion of incident T2DM events occurring within 2 years, cause‐specific and Fine–Gray competing‐risk analyses accounting for death, exclusion of participants with baseline prediabetes, and a multiple‐imputation sensitivity analysis including all incomplete covariates. Prediabetes was defined according to American Diabetes Association criteria as fasting plasma glucose 5.6–6.9 mmol/L or glycated haemoglobin (HbA1c) 5.7%–6.4% (39–46 mmol/mol). For this sensitivity analysis, 20 imputed datasets were generated using multiple imputation by chained equations (MICE), with all covariates, the outcome indicator, and Nelson–Aalen cumulative hazard estimates included in the imputation model. Logistic or multinomial regression and predictive mean matching were applied according to variable type, and estimates were pooled using Rubin's rules. Convergence and distributional diagnostics were assessed.

All analyses were performed using R statistical software, version 4.5.1 (R Foundation for Statistical Computing). All tests were two‐sided; *p* < 0.05 indicated statistical significance, and 95% CIs were reported.

## 3 Results

Participant characteristics: Baseline characteristics of the 60,249 participants, with mean age of 54.5 ± 7.5 years, stratified by poor, intermediate, and healthy sleep patterns, are shown in Table 1. Most participants had healthy sleep (57.6%), whereas 1352 (2.2%) had poor sleep. Participants with healthy sleep were more likely to be female, white, have higher household income, live in areas with a lower TDI, never smoke, consume light or moderate amounts of alcohol, and be physically active. In contrast, participants with poor sleep had a higher cardiometabolic burden, including a higher BMI and TyG index and a greater prevalence of hypertension and hyperlipidaemia. Characteristics of the study population based on T2DM status are presented in Supporting Information S1: Table S3. During a median follow‐up of 4.2 years, 1284 (2.1%) individuals were diagnosed with T2DM. Compared to the non‐T2DM group, participants with T2DM were older, more likely to be male (60.9%), and faced greater socioeconomic disadvantage. The T2DM sub‐cohort also had unhealthier lifestyle behaviours, such as higher rates of physical inactivity and smoking, and a significantly increased burden of comorbidities, including higher BMI, hypertension (41.7%), and an elevated TyG index.

**TABLE 1:** Baseline characteristics of participants based on sleep patterns at baseline.

	Total	Sleep patterns			*p* value
		Poor	Intermediate	Healthy	
*n*	60,249	1352	24,182	34,715	
Age, years	54.5 ± 7.5	54.8 ± 7.1	54.9 ± 7.4	54.2 ± 7.6	< 0.001
Sex, male	28,298 (47.0%)	675 (49.9%)	12,332 (51.0%)	15,291 (44.0%)	< 0.001
Ethnicity, white	58,681 (97.4%)	1288 (95.3%)	23,514 (97.2%)	33,879 (97.6%)	< 0.001
TDI	−1.9 ± 2.7	−1.4 ± 2.9	−1.8 ± 2.8	−2.0 ± 2.7	< 0.001
University degree					< 0.001
Degree‐level	27,927 (46.4%)	704 (52.1%)	11,568 (47.8%)	15,655 (45.1%)	
Non‐college	27,622 (45.8%)	481 (35.6%)	10,370 (42.9%)	16,771 (48.3%)	
Missing	4700 (7.8%)	167 (12.4%)	2244 (9.3%)	2289 (6.6%)	
Household income					< 0.001
< £18,000	5910 (9.8%)	210 (15.5%)	2659 (11.0%)	3041 (8.8%)	
£18,000–£51,999	28,088 (46.6%)	653 (48.3%)	11,275 (46.6%)	16,160 (46.6%)	
> £52,000	20,422 (33.9%)	343 (25.4%)	7594 (31.4%)	12,485 (36.0%)	
Missing	5829 (9.7%)	146 (10.8%)	2654 (11.0%)	3029 (8.7%)	
Smoking status					< 0.001
Never	36,946 (61.3%)	740 (54.7%)	14,033 (58.0%)	22,173 (63.9%)	
Current	3832 (6.4%)	140 (10.4%)	1881 (7.8%)	1811 (5.2%)	
Previous	19,471 (32.3%)	472 (34.9%)	8268 (34.2%)	10,731 (30.9%)	
Alcohol consumption status					< 0.001
Abstainer	771 (1.3%)	30 (2.2%)	316 (1.3%)	425 (1.2%)	
Light or moderate	36,487 (60.6%)	707 (52.3%)	13,987 (57.8%)	21,793 (62.8%)	
Heavy or abusive	12,813 (21.3%)	332 (24.6%)	5885 (24.3%)	6596 (19.0%)	
Missing	10,178 (16.9%)	283 (20.9%)	3994 (16.5%)	5901 (17.0%)	
Physical activity					< 0.001
Never	1999 (3.3%)	121 (8.9%)	1062 (4.4%)	816 (2.4%)	
Low activity	1426 (2.4%)	50 (3.7%)	706 (2.9%)	670 (1.9%)	
Medium activity	47,762 (79.3%)	1051 (77.7%)	19,240 (79.6%)	27,471 (79.1%)	
High activity	9062 (15.0%)	130 (9.6%)	3174 (13.1%)	5758 (16.6%)	
BMI, kg/m^2^	26.5 ± 4.2	28.4 ± 4.8	27.1 ± 4.3	26.0 ± 3.9	< 0.001
Hypertension	12,179 (20.2%)	401 (29.7%)	5634 (23.3%)	6144 (17.7%)	< 0.001
Hyperlipidaemia	4273 (7.1%)	152 (11.2%)	1924 (8.0%)	2197 (6.3%)	< 0.001
TyG index	8.6 ± 0.5	8.8 ± 0.5	8.7 ± 0.5	8.6 ± 0.5	< 0.001

*Note:* Continuous variables are expressed as mean ± standard deviation, and categorical variables are expressed as *n* (%).

Abbreviations: BMI, body mass index; TDI, Townsend deprivation index; TyG, triglyceride‐glucose index.

Sleep patterns at individual visits and T2DM risk: The association between sleep patterns and the risk of T2DM is presented in Supporting Information S1: Table S4. In the fully adjusted Model, healthy sleep at baseline was associated with a 47% lower risk of T2DM compared with poor sleep (HR: 0.53; 95% CI: 0.41, 0.68). Each 1‐point increase in the HSS corresponded to a 14% reduction in risk (HR: 0.86; 95% CI: 0.82, 0.91). Similar associations were observed at the second visit, where healthy sleep patterns were associated with a significantly lower risk of T2DM (HR: 0.66; 95% CI: 0.51, 0.85) compared with the poor sleep group.

Sleep pattern transitions and risk of T2DM: The association of sleep‐pattern transitions is shown in Table 2. Compared to the stable healthy group, participants with stable poor sleep had a more than two‐fold risk of T2DM (HR: 2.07; 95% CI: 1.32, 3.27). Within the entire cohort, both worsening (HR: 1.43) and optimising (HR: 1.41) sleep patterns were associated with a higher risk compared with the stable healthy group. In contrast, transitions towards better sleep categories were not associated with a reduced risk of T2DM compared with those remaining in the same baseline category. The fully adjusted HRs were 0.95 (95% CI, 0.55–1.63) for poor‐to‐intermediate sleep, 1.20 (95% CI, 0.49–2.93) for poor‐to‐healthy sleep, and 1.00 (95% CI, 0.84–1.21) for intermediate‐to‐healthy sleep (Supporting Information S1: Table S5).

**TABLE 2:** Associations between sleep pattern changes from baseline to follow‐up and the risk of T2DM by baseline sleep status.

Sleep pattern changes	Cases/*N*	HR (95% CI)		
		Model 1	Model 2	Model 3
Entire participants	1284/60,249	—	—	—
Stable poor	21/354	3.45 (2.22, 5.36)	2.16 (1.39, 3.37)	2.07 (1.32, 3.27)
Worsening	262/11,680	1.66 (1.41, 1.95)	1.44 (1.22, 1.68)	1.43 (1.21, 1.68)
Stable intermediate	394/15,216	1.68 (1.45, 1.93)	1.33 (1.15, 1.53)	1.31 (1.13, 1.52)
Optimising	250/8753	1.84 (1.57, 2.17)	1.48 (1.26, 1.75)	1.41 (1.20, 1.67)
Stable healthy	357/24,246	*Ref*	*Ref*	*Ref*
Healthy sleep pattern at baseline	577/34,715	—	—	—
Stable healthy	357/24,246	*Ref*	*Ref*	*Ref*
Worsening	220/10,469	1.44 (1.21, 1.70)	1.29 (1.09, 1.52)	1.41 (1.19, 1.68)
Non‐healthy sleep pattern at baseline^a^	707/25,534	—	—	—
Non‐worsening^b^	644/23,969	*Ref*	*Ref*	*Ref*
Non‐improvement^c^	63/1565	1.74 (1.42, 2.13)	1.38 (1.13, 1.70)	1.28 (1.03, 1.58)

*Note:* Model 1 was adjusted for age, sex, and ethnicity. Model 2 was adjusted for age, sex, ethnicity, household income, TDI, education level, employment status, smoking status, alcohol consumption, physical activity, and obesity. Model 3 was adjusted for age, sex, ethnicity, household income, TDI, education level, employment status, smoking status, alcohol consumption, physical activity, obesity, history of hypertension, history of hyperlipidaemia, and the PRS for diabetes.

Abbreviations: CI, confidence interval; HR, hazard ratio; PRS, polygenic risk score; TDI, Townsend deprivation index; T2DM, type 2 diabetes mellitus.

^a^ Non‐healthy sleep pattern at baseline refers to a baseline sleep pattern that is intermediate or poor.

^b^ Non‐worsening, including optimising and stable intermediate from baseline.

^c^ Non‐improvement, including worsening and stable poor from baseline.

As shown in Table 2 and Supporting Information S1: Table S5, analysis based on baseline status revealed that among participants starting with a healthy sleep pattern, participants with worsening patterns had a significantly higher risk compared to the stable healthy group (HR: 1.41; 95% CI: 1.19, 1.68); while within a non‐healthy sleep pattern, those who failed to show improvement (non‐improvement) had a significantly higher risk compared to those who optimised their sleep (HR: 1.28; 95% CI: 1.03, 1.58). Furthermore, in the baseline intermediate group, a shift towards worsening sleep was associated with an HR of 1.13, whereas in the baseline healthy group, even minor worsening significantly elevated the risk (HR: 1.40). These results remained consistent in sensitivity analyses excluding cases diagnosed within 2 years (Supporting Information S1: Table S6).

Supporting Information S1: Table S7 presents person‐years, incidence rates, model‐standardized four‐year risks, and risk differences across the five pooled sleep‐transition categories. Incidence rates ranged from 3.62 per 1000 person‐years (95% CI, 3.25–4.02) among participants with stable healthy sleep to 14.14 per 1000 person‐years (95% CI, 8.76–21.62) among those with stable poor sleep. The corresponding model‐standardized four‐year risks were 1.06% (95% CI, 0.94%–1.20%) and 2.13% (95% CI, 1.29%–2.93%), respectively, yielding an absolute risk difference of 1.07 percentage points (95% CI, 0.22–1.87).

Changes in sleep scores and dose‐response relationships: Analysis of signed changes in HSS demonstrated that both decreases and increases in the score were associated with risk (Supporting Information S1: Table S8). Relative to no change, the HRs were 1.14 (95% CI, 1.00–1.30; *P* = 0.049) for a decrease and 1.14 (95% CI, 1.00–1.31; *P* = 0.049) for an increase, while the linear estimate per one‐point increase was null (HR, 0.99; 95% CI, 0.94–1.05). Restricted cubic splines (Figure 1A) showed a U‐shaped dose‐response relationship for the total population, indicating that stable healthy sleep was associated with the lowest risk. Participant and event counts across signed HSS‐change values are provided in Supporting Information S1: Table S9.

**FIGURE 1:**
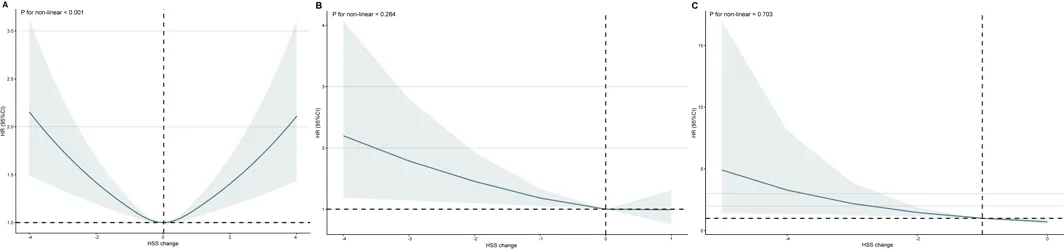
Association between signed change in healthy sleep score from visit 1 to visit 2 and incident T2DM. Panels show (A) all participants, (B) participants with baseline HSS of 4, and (C) participants with baseline HSS of 5. Restricted cubic spline models were adjusted using the prespecified Model 3 covariates. Solid lines represent hazard ratios, and shaded areas indicate 95% confidence intervals. Panel A used knots at signed HSS changes of −1, 0, and 1, with 0 as the reference (*N* = 60,249; 1284 events); both the overall association and nonlinearity tests were *p* < 0.001. Panel B used knots at −1, 0, and 1, with 0 as the reference (*N* = 22,769; 417 events); the overall association *p* value was 0.034 and the nonlinearity *p* value was 0.284. Panel C used knots at −2, −1, and 0, with −1 as the reference (*N* = 11,946; 160 events); the overall association *p* value was < 0.001 and the nonlinearity *p* value was 0.703. HR, hazard ratio; HSS, healthy sleep score; T2DM, type 2 diabetes mellitus.

However, this relationship was modified by the baseline HSS. In participants with high baseline scores (4–5), the risk decreased linearly as the positive change in sleep score increased (Figure 1B and 1C, Supporting Information S1: Figure S3B). In contrast, those with low baseline scores (0–3) showed an approximately linear increase in risk as the HSS changed (Supporting Information S1: Figure S3A). These results remained consistent in sensitivity analyses excluding cases diagnosed within two years (Supporting Information S1: Table S10 and Figure S4).

Individual sleep dimensions and subgroup analyses: Associations between changes in individual sleep dimensions and T2DM risk are presented in Supporting Information S1: Table S11. Stable unhealthy sleep duration (HR, 1.35), insomnia (HR, 1.24), and snoring (HR, 1.24) were associated with higher risk. A transition from morning to evening chronotype (HR, 1.31) and new‐onset insomnia (HR, 1.39) or daytime dozing (HR, 1.38) were also associated with higher risk.

Exploratory subgroup analyses are shown in Figure 2 and Supporting Information S1: Table S12. HRs represent a one‐category increase in the ordered sleep‐transition code. Only age remained significant after Benjamini–Hochberg correction (*P* = 0.005; *q* = 0.044); no other subgroup interaction remained significant.

**FIGURE 2:**
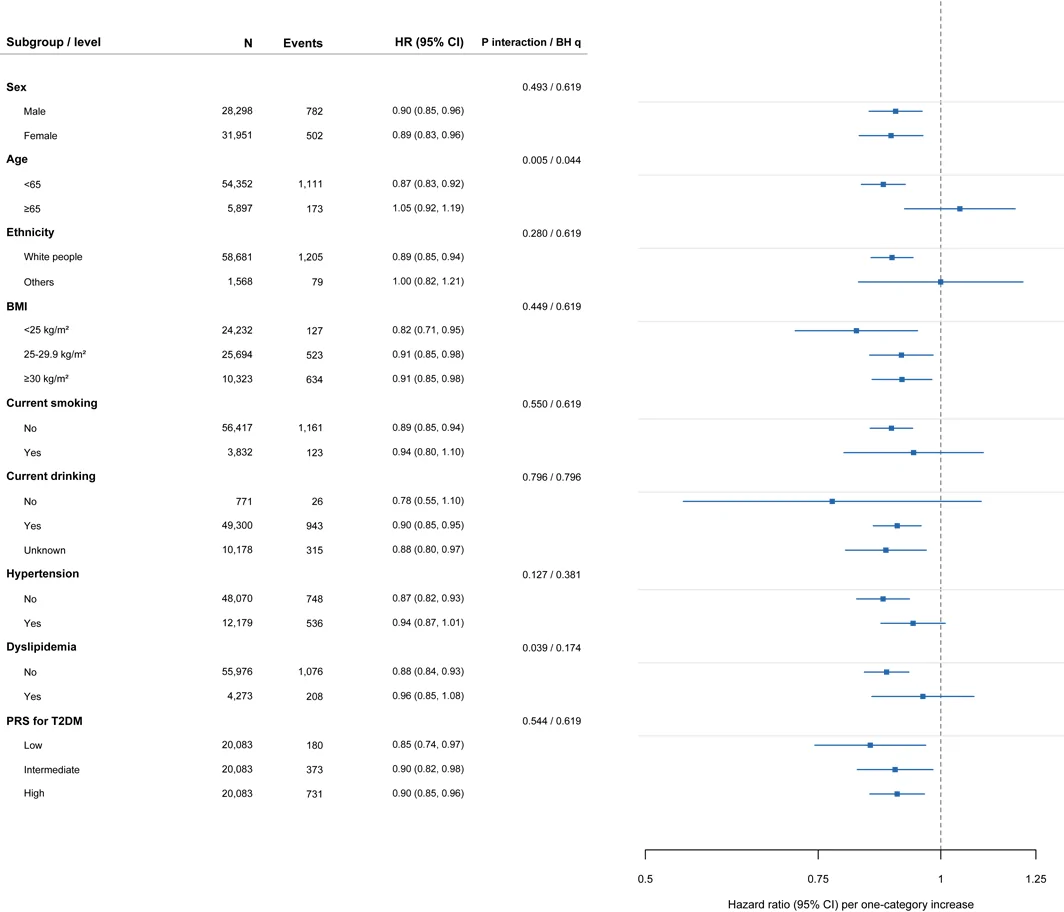
Subgroup analyses of the ordered sleep‐transition code and incident T2DM. Squares and horizontal lines indicate HRs and 95% CIs per one‐category increase. *N*, events, interaction *p* values, and Benjamini–Hochberg *q* values are shown. BMI, body mass index; PRS, polygenic risk score; T2DM, type 2 diabetes mellitus.

Sensitivity analyses: Sensitivity analyses produced estimates that were consistent with the primary findings. In the complete‐case analysis (58,544 participants; 1234 events), the HRs versus stable healthy sleep were 2.05 (95% CI, 1.30–3.23) for stable poor sleep, 1.42 (95% CI, 1.20–1.67) for worsening sleep, 1.30 (95% CI, 1.12–1.51) for stable intermediate sleep, and 1.41 (95% CI, 1.19–1.67) for optimising sleep (Supporting Information S1: Table S13). Alternative linear and nonlinear BMI specifications (Supporting Information S1: Table S14), exclusion of events occurring within two years (Supporting Information S1: Tables S6 and S10), cause‐specific and Fine–Gray competing‐risk analyses (Supporting Information S1: Tables S15 and S16), and exclusion of participants with baseline prediabetes (Supporting Information S1: Table S17) yielded similar estimates. The global Schoenfeld residual test did not indicate an overall violation of the proportional‐hazards assumption (*P* = 0.127), although the transition‐term test had *P* = 0.041 (Supporting Information S1: Table S18). In the multiple‐imputation sensitivity analysis including all incomplete covariates, the Model 3 HRs relative to no HSS change were 1.151 (95% CI, 1.010–1.313) for a decrease and 1.149 (95% CI, 1.005–1.314) for an increase (Supporting Information S1: Table S19).

Exploratory analysis of the TyG index: In fully adjusted linear regression models, sleep transition categories were associated with differences in the TyG index (Supporting Information S1: Table S20). Compared with participants with stable healthy sleep, those with stable poor sleep, stable intermediate sleep, worsening sleep, and optimising sleep patterns had higher TyG indices, with *β* values of 0.097 (95% CI, 0.040–0.154), 0.053 (0.042–0.064), 0.020 (0.008–0.032), and 0.035 (0.022–0.049), respectively (all *p* < 0.001). In analyses stratified by baseline sleep status, improvement from intermediate to healthy sleep was associated with a lower TyG index (β, −0.026; 95% CI, −0.041 to −0.011; *p* < 0.001), whereas transitions from poor sleep to better categories were not significantly associated with TyG levels.

## 4 Discussion

The present study examined sleep‐pattern transitions between two assessments and incident T2DM. At each assessment, a healthy sleep pattern was associated with lower T2DM risk, consistent with previous large prospective studies [17, 18]. Beyond single‐point assessments, the pooled transition analysis showed that stable poor, worsening, and optimising groups had higher risk than the stable healthy group. This is consistent with evidence that repeated sleep assessments may provide information beyond sleep quality at a single time point [19, 20]. This study observed a U‐shaped relationship between the change in HSS and T2DM risk across the entire population. This nonlinear trend suggests that departures in either direction from no change were associated with increased risk. While the association between declining sleep quality and diabetes risk is well‐documented [17, 18], our observation of elevated risk in the optimising group suggests that an upward shift in sleep quality may not immediately reverse the pre‐existing risk. This contrasts with a smaller intervention study that reported rapid metabolic improvements following sleep enhancement [21], suggesting that in large‐scale, real‐world populations, associations with prior poor sleep may persist.

Several biological pathways may link sleep patterns to glucose metabolism. Primary among these is circadian disruption, particularly observed in individuals transitioning chronotype from morning to evening. This desynchronisation between the ‘central clock’ in the suprachiasmatic nucleus and ‘peripheral clocks’ in the pancreas or liver impairs *β*‐cell function and reduces insulin secretion [22, 23]. Furthermore, poor sleep quality, characterised by fragmentation and insomnia, may chronically activate the sympathetic nervous system and the hypothalamic‐pituitary‐adrenal axis, which may contribute to insulin resistance [24]. Additionally, sleep disturbances such as snoring and daytime dozing may contribute to systemic inflammation and oxidative stress. Intermittent hypoxia increases the production of pro‐inflammatory cytokines such as tumour necrosis factor‐alpha (TNF‐α) and interleukin‐6 (IL‐6), which interfere with insulin signalling pathways [25]. Finally, sleep deprivation or poor sleep quality alters the neuroendocrine control of appetite, significantly decreasing leptin levels while increasing ghrelin. This hormonal imbalance may promote hyperphagia and weight gain, which may contribute to the metabolic burden and progression towards T2DM [26, 27].

Our exploratory analysis of the TyG index provides additional information on metabolic correlates of sleep transitions with T2DM risk. The TyG index, a validated surrogate marker of insulin resistance, reflects combined abnormalities in glucose and lipid metabolism and may reflect early metabolic disturbances associated with sleep disruption and circadian dysregulation [17, 28]. Compared with stable healthy sleep, most unfavourable sleep transition categories, including stable poor sleep, stable intermediate sleep, worsening sleep, and even optimising sleep patterns, were associated with higher TyG levels. These findings indicate that sleep‐pattern transitions were associated with metabolic differences that persisted despite reported sleep improvement. Among participants whose sleep improved, the TyG index remained higher than that of the stable healthy group, which may reflect residual metabolic differences or the influence of earlier sleep patterns [29, 30]. These findings are consistent with a role for impaired glucose–lipid metabolism in the association between sleep‐pattern transitions and T2DM risk.

When stratified by baseline HSS, the U‐shaped pattern was not uniform. Among participants with high baseline scores (4–5), higher HSS change was linearly associated with lower T2DM risk. This suggests that for those with healthy baseline sleep, stability and incremental improvement were associated with reduced risk, consistent with evidence that the benefits of sleep quality maintenance may accrue from early intervention [31]. In participants with low baseline scores (0–3), the association was approximately linear in the opposite direction. This divergence suggests that the interpretation of sleep score change depends on baseline sleep status: for those with healthy sleep, maintenance or improvement tracked with lower risk; for those with poor sleep, score change may signal a more complex risk profile that a single behavioural change does not fully address. These findings underscore the relevance of repeated sleep assessment in epidemiologic studies of diabetes.

Analysis of individual sleep dimensions showed associations between sleep‐pattern transitions and metabolic health. Across most domains, transitions away from healthy baseline states—even when representing apparent improvements—were associated with higher T2DM risk, suggesting that changes in sleep patterns may disrupt circadian and metabolic regulation. Notably, the impact of sleep worsening appeared to be asymmetric. New‐onset insomnia was associated with a higher risk than stable insomnia, suggesting that acute sleep disruption may be associated with greater metabolic stress than long‐standing sleep problems. Similarly, transitioning in chronotype was associated with increased risk, whereas a stable evening chronotype was not, suggesting that chronotype transitions may be more strongly associated with metabolic risk than an inherent evening preference [22]. The potential for risk attenuation after improvement varied across sleep dimensions, providing insights into possible intervention opportunities. Sleep duration showed the greatest potential for recovery, with optimization bringing risk estimates closer to those of stable healthy sleep, consistent with lower risk after reported improvement in sleep duration [32, 33]. In contrast, daytime dozing emerged as one of the factors most strongly associated with T2DM. Its persistent association with risk suggests that excessive daytime sleepiness may reflect underlying systemic inflammation or hypothalamic dysregulation rather than simply poor sleep behaviour [34, 35, 36]. Reported snoring relief was associated with a higher risk than those with stable snoring. This may reflect heterogeneity in sleep‐disordered breathing or reporting, consistent with evidence that self‐reported snoring correlates poorly with obstructive sleep apnoea (OSA) severity [37, 38]. The subgroup analyses showed that age modified the relationship between changes in sleep patterns and T2DM risk. The stronger association in younger participants (< 65 years) may reflect greater susceptibility to sleep‐related metabolic disturbance in mid‐life. Because these analyses involved multiple comparisons and some categories had limited sample sizes, the findings should be interpreted as hypothesis‐generating.

Several limitations should be considered. First, sleep parameters were assessed using self‐reported questionnaires rather than objective measures such as polysomnography or actigraphy. Therefore, measurement errors due to recall bias and the inability to capture sleep microarchitecture or asymptomatic sleep‐disordered breathing cannot be excluded. Second, sleep was assessed at only two time points; trajectories, regularity, and sustained changes could not be examined because of the public data. Third, although we adjusted for a broad range of potential confounders, residual confounding from unmeasured factors, including changes in psychological stress and other time‐varying characteristics, may remain. Fourth, TyG was measured at visit 1, before the sleep transition interval, and repeated visit‐2 triglyceride and glucose measurements were unavailable; therefore, change in TyG and mediation could not be evaluated, and the TyG analyses were treated as exploratory. Fifth, glucose and HbA1c were unavailable at visit 2, so prediabetes at that visit could not be defined; the available sensitivity analysis excluded baseline prediabetes. Sixth, component and subgroup analyses involved multiple comparisons, and some categories had limited sample sizes; therefore, these findings should be interpreted as hypothesis‐generating. In addition, requiring participants to survive and remain diabetes‐free until visit 2 to enter the landmark cohort may have introduced selection bias. Finally, the UK Biobank cohort predominantly comprises individuals of European ancestry and is subject to healthy volunteer bias, which may limit the generalisability of our findings to populations with greater ethnic and socioeconomic diversity or different baseline sleep‐metabolic profiles.

## 5 Conclusions

In this cohort, sleep pattern transitions between the two assessments were associated with incident type 2 diabetes. Stable poor sleep carried the highest risk, and worsening of healthy sleep was associated with elevated risk. The U‐shaped relationship between sleep score change and diabetes risk suggests that both deterioration and large changes in self‐reported sleep may be associated with increased risk. These findings indicate that repeated sleep assessment captures information beyond a single measurement in relation to diabetes risk.

## Author Contributions

C.G., Y.Z., and Z.C. designed the research. Z.C. and Y.Z. performed the statistical analysis. S.Z., Y.G., Q.G., F.Z., Y.C., F.L., and K.D. contributed to data collection and interpretation. Y.Z. and Z.C. wrote the paper. Z.C., Y.Z., F.L., K.D., and C.G. revised the manuscript. All authors have read and approved the final manuscript. Z.C. is the guarantor of this work and, as such, had full access to all the data in the study and takes responsibility for the integrity of the data and the accuracy of the data analysis.

## Funding

This work was supported by Beijing Natural Science Foundation (JQ24039), Noncommunicable Chronic Diseases‐National Science and Technology Major Project (2025ZD0548201), and CAMS Innovation Fund for Medical Sciences (2024‐I2M‐3‐020).

## Ethics Statement

The Northwest Multi‐Centre Research Ethics Committee approved the UKB study initially in 2011 (REC reference number for UK Biobank: 11/NW/0382), and this approval was renewed in 2016 and 2021.

## Consent

Written informed consent was obtained from all participants.

## Conflicts of Interest

The authors declare no conflicts of interest.

## Supporting information


Supporting Information S1

## Data Availability

Researchers interested in accessing the data used in this study can apply for access to the UK Biobank by visiting their website (https://www.ukbiobank.ac.uk/enable‐your‐research/apply‐for‐access) and submitting an application that includes a research protocol summary and requested data fields. Upon approval by the UK Biobank management team and payment of applicable fees, researchers will be granted access to the dataset.
