# Supplementary material for: Longitudinal Sleep Patterns and Risk of Type 2 Diabetes Mellitus
Source: Diabetes Metab Res Rev. 2026 Oct 12;42(7):e70237. doi: 10.1002/dmrr.70237 (PMC13656764; doi:10.1002/dmrr.70237)
Supplement: Supplementary file 1 [file DMRR-42-e70237-s001.docx]

**Longitudinal Sleep Patterns and Risk of Type 2 Diabetes Mellitus**

Ying Zhang, MD^1#^, Zhen’ge Chang, MD^2#^, Shuang Zhang, MD^2^, Yingying Guo, MD^2^, Qian Guo, MD^3^, Feiyu Zhang, MD^3^, Yating Chen, MD^3^, Fang Luo, MD^2*^, Kefei Dou, MD^2,4*^, Caixia Guo, MD^1*^

**Affiliations:**

^1^ Cardiovascular Center, Beijing Tongren Hospital, Capital Medical University, Beijing, China

^2^ Cardiometabolic Medicine Center, Fuwai Hospital, National Center for Cardiovascular Diseases, Chinese Academy of Medical Sciences & Peking Union Medical College, Beijing, China

^3^ Center for Coronary Artery Disease, Division of Cardiology, Beijing Anzhen Hospital, Capital Medical University, Beijing, China

^4^ State Key Laboratory of Cardiovascular Disease, Beijing, China

#Ying Zhang and Zhen’ge Chang contributed equally to this work.

**^*^Correspondence to:**

Caixia Guo, MD, Cardiovascular Center, Beijing Tongren Hospital, Capital Medical University, Beijing, China. E-mail: cxgbb@163.com

Kefei Dou, MD, Cardiometabolic Medicine Center, Fuwai Hospital, National Center for Cardiovascular Diseases, Chinese Academy of Medical Sciences & Peking Union Medical College, Beijing, China. E-mail: drdoukefei@126.com

Fang Luo, MD, Cardiometabolic Medicine Center, Fuwai Hospital, National Center for Cardiovascular Diseases, Chinese Academy of Medical Sciences & Peking Union Medical College, Beijing, China. E-mail: luofang@fuwaihospital.org

**Supplementary Appendix**

**Supplementary Table S1. Field IDs of the UK Biobank Variables Used in the Study.**

| **Variables** | **Field ID/ICD-10 codes** |
| --- | --- |
| Sleep data | Field ID 1160/1180/1200/1210/1220 |
| Age | Field ID 21003 |
| Sex | Field ID 31 |
| Ethnicity | Field ID 21000 |
| Townsend deprivation index | Field ID 22189 |
| Household income | Field ID 738 |
| Current employment status | Field ID 6142 |
| Education level | Field ID 6138 |
| Smoking status | Field ID 20116 |
| Drinking status | Field ID 20117 |
| Physical activity | Field ID 22033 |
| Body mass index | Field ID 21001 |
| Glucose | Field ID 30740 |
| TG | Field ID 30870 |
| HbA1c | Field 30750 |
| PRS for T2DM | Field ID 26285 |
| Cardiovascular disease | Field ID 6150, ICD I00-I99 |
| Hypertension | Field ID 6150, ICD I10-I15 |
| Hyperlipidemia | Field ID 135, ICD E78 |
| Incident T2DM after visit 2 | First linked ICD-10 E11 record after the visit-2 landmark |
| Prevalent T1DM (n=372) | Self-reported doctor diagnosis (Field 2443) or linked ICD-10 E10 before visit 2 |
| Prevalent T2DM (n=5,563) | Self-reported doctor diagnosis (Field 2443) or linked ICD-10 E11 before visit 2 |
| Laboratory-defined diabetes | Glucose ≥7.0 mmol/L (Field 30740) or HbA1c ≥6.5%/48 mmol/mol (Field 30750) |
| Diabetes medication use | Fields 20003, 6153, or 6177 |

Abbreviations: HbA1c, glycated hemoglobin; ICD-10, International Classification of Diseases, Tenth Revision; PRS, polygenic risk score; T1DM, type 1 diabetes mellitus; T2DM, type 2 diabetes mellitus; TG, triglyceride.

**Supplementary Table S2. Changes in Sleep Patterns Between Visit 1 (2006–2010) and Visit 2 (2014+).**

| **Sleep patterns at two assessments** | | **Sleep-pattern transition category** |
| --- | --- | --- |
| **Assessment visit 1 (2006–2010)** | **Assessment visit 2 (2014+)** |  |
| Poor sleep pattern | Poor sleep pattern | Stable poor sleep pattern |
|  | Intermediate sleep pattern | Optimizing sleep pattern |
|  | Healthy sleep pattern | Optimizing sleep pattern |
| Intermediate sleep pattern | Poor sleep pattern | Worsening sleep pattern |
|  | Intermediate sleep pattern | Stable intermediate sleep pattern |
|  | Healthy sleep pattern | Optimizing sleep pattern |
| Healthy sleep pattern | Poor sleep pattern | Worsening sleep pattern |
|  | Intermediate sleep pattern | Worsening sleep pattern |
|  | Healthy sleep pattern | Stable healthy sleep pattern |

**Supplementary Table S3. Baseline Characteristics of Participants Based on** **Diabetes Mellitus Status.**

| **Characteristic** | **Total** | **Non-T2DM** | **T2DM** | ***P* Value** |
| --- | --- | --- | --- | --- |
| n | 60,249 | 58,965 | 1,284 |  |
| Age, years | 54.5 ± 7.5 | 54.5 ± 7.5 | 56.1 ± 7.4 | <0.001 |
| Sex, male | 28,298 (47.0%) | 27,516 (46.7%) | 782 (60.9%) | <0.001 |
| Ethnicity, white | 58,681 (97.4%) | 57,476 (97.5%) | 1,205 (93.8%) | <0.001 |
| Townsend deprivation index | -1.9 ± 2.7 | -1.9 ± 2.7 | -1.4 ± 2.9 | <0.001 |
| University degree |  |  |  | <0.001 |
| Degree-level education | 27,927 (46.4%) | 27,251 (46.2%) | 676 (52.6%) |  |
| Non-college education | 27,622 (45.8%) | 27,168 (46.1%) | 454 (35.4%) |  |
| Missing | 4,700 (7.8%) | 4,546 (7.7%) | 154 (12.0%) |  |
| Household income |  |  |  | <0.001 |
| <£18,000 | 5,910 (9.8%) | 5,724 (9.7%) | 186 (14.5%) |  |
| £18,000–£51,999 | 28,088 (46.6%) | 27,431 (46.5%) | 657 (51.2%) |  |
| >£52,000 | 20,422 (33.9%) | 20,120 (34.1%) | 302 (23.5%) |  |
| Missing | 5,829 (9.7%) | 5,690 (9.6%) | 139 (10.8%) |  |
| Smoking status |  |  |  | <0.001 |
| Never | 36,946 (61.3%) | 36,277 (61.5%) | 669 (52.1%) |  |
| Current | 3,832 (6.4%) | 3,709 (6.3%) | 123 (9.6%) |  |
| Previous | 19,471 (32.3%) | 18,979 (32.2%) | 492 (38.3%) |  |
| Alcohol consumption status |  |  |  | <0.001 |
| Abstainer | 771 (1.3%) | 745 (1.3%) | 26 (2.0%) |  |
| Light or moderate | 36,487 (60.6%) | 35,809 (60.7%) | 678 (52.8%) |  |
| Heavy or abusive | 12,813 (21.3%) | 12,548 (21.3%) | 265 (20.6%) |  |
| Missing | 10,178 (16.9%) | 9,863 (16.7%) | 315 (24.5%) |  |
| Physical activity |  |  |  | <0.001 |
| Never | 1,999 (3.3%) | 1,897 (3.2%) | 102 (7.9%) |  |
| Low activity | 1,426 (2.4%) | 1,372 (2.3%) | 54 (4.2%) |  |
| Medium activity | 47,762 (79.3%) | 46,739 (79.3%) | 1,023 (79.7%) |  |
| High activity | 9,062 (15.0%) | 8,957 (15.2%) | 105 (8.2%) |  |
| BMI, kg/m^2^ | 26.5 ± 4.2 | 26.4 ± 4.1 | 30.9 ± 5.3 | <0.001 |
| Hypertension | 12,179 (20.2%) | 11,643 (19.7%) | 536 (41.7%) | <0.001 |
| Hyperlipidemia | 4,273 (7.1%) | 4,065 (6.9%) | 208 (16.2%) | <0.001 |
| TyG index | 8.6 ± 0.5 | 8.6 ± 0.5 | 9.2 ± 0.6 | <0.001 |

Continuous variables are expressed as mean ± standard deviation, and categorical variables are expressed as n (%).

Abbreviations: BMI, body mass index; T2DM, type 2 diabetes mellitus; TyG, triglyceride-glucose index.

**Supplementary Table S4. Association between Sleep Patterns and the Risk of T2DM at Baseline and Follow-up.**

| **Exposure** | **Cases/N** | **HR (95% CI)** | | | **P for trend** |
| --- | --- | --- | --- | --- | --- |
|  |  | **Model 1** | **Model 2** | **Model 3** |  |
| **Visit 1** |  |  |  |  |  |
| Poor | 75/1,352 | Reference | Reference | Reference | <0.001 |
| Intermediate | 632/24,182 | **0.47 (0.37, 0.60)** | **0.61 (0.48, 0.77)** | **0.64 (0.50, 0.83)** |  |
| Healthy | 577/34,715 | **0.32 (0.25, 0.40)** | **0.49 (0.39, 0.63)** | **0.53 (0.41, 0.68)** |  |
| Per 1 score increase | 1,284/60,249 | **0.76 (0.72, 0.80)** | **0.85 (0.81, 0.90)** | **0.86 (0.82, 0.91)** |  |
| **Visit 2** |  |  |  |  |  |
| Poor | 70/1,844 | Reference | Reference | Reference | <0.001 |
| Intermediate | 654/26,253 | **0.65 (0.50, 0.83)** | **0.77 (0.60, 0.98)** | 0.81 (0.63, 1.04) |  |
| Healthy | 560/32,152 | **0.45 (0.35, 0.57)** | **0.62 (0.48, 0.80)** | **0.66 (0.51, 0.85)** |  |
| Per 1 score increase | 1,284/60,249 | **0.79 (0.75, 0.83)** | **0.87 (0.83, 0.92)** | **0.88 (0.83, 0.93)** |  |

Model 1 was adjusted for age, sex, and ethnicity.

Model 2 was adjusted for age, sex, ethnicity, household income, TDI, education level, employment status, smoking status, alcohol consumption, physical activity, and obesity.

Model 3 was adjusted for age, sex, ethnicity, household income, TDI, education level, employment status, smoking status, alcohol consumption, physical activity, obesity, history of hypertension, history of hyperlipidemia, and the PRS for T2DM.

Significant P values are in bold.

Abbreviations: CI, confidence interval; HR, hazard ratio; T2DM, type 2 diabetes mellitus; TDI, Townsend deprivation index; PRS, polygenic risk score.

**Supplementary Table S5. Associations Between Sleep Pattern Changes from Baseline to Follow-Up and the Risk of T2DM by Baseline Sleep Status.**

| **Sleep pattern changes** | **Cases/N** | **Incidence/1,000 PY (95% CI)** | **HR (95% CI)** | | |
| --- | --- | --- | --- | --- | --- |
|  |  |  | **Model 1** | **Model 2** | **Model 3** |
| **Poor sleep pattern at baseline** | 75/1,352 |  |  |  |  |
| Stable poor | 21/354 | 14.14 (8.76, 21.62) | *Ref* | *Ref* | *Ref* |
| Moderate optimizing | 47/847 | 13.70 (10.07, 18.22) | 0.98 (0.59, 1.65) | 0.96 (0.56, 1.64) | 0.95 (0.55, 1.63) |
| Substantial optimizing | 7/151 | 12.02 (4.83, 24.77) | 0.92 (0.39, 2.17) | 1.10 (0.46, 2.64) | 1.20 (0.49, 2.93) |
| **Intermediate sleep pattern at baseline** | 632/24,182 |  |  |  |  |
| Stable intermediate | 394/15,216 | 6.38 (5.77, 7.04) | *Ref* | *Ref* | *Ref* |
| Worsening | 42/1,211 | 8.85 (6.38, 11.96) | **1.43 (1.04, 1.97)** | 1.22 (0.89, 1.68) | 1.13 (0.82, 1.56) |
| Optimizing | 196/7,755 | 6.28 (5.43, 7.22) | 0.98 (0.83, 1.17) | 0.99 (0.83, 1.19) | 1.00 (0.84, 1.21) |
| **Healthy sleep pattern at baseline** | 577/34,715 |  |  |  |  |
| Stable healthy | 357/24,246 | 3.62 (3.25, 4.02) | *Ref* | *Ref* | *Ref* |
| Moderate worsening | 213/10,190 | 5.46 (4.75, 6.24) | **1.55 (1.31, 1.84)** | **1.38 (1.17, 1.64)** | **1.40 (1.18, 1.67)** |
| Substantial worsening | 7/279 | 7.21 (2.90, 14.86) | **2.15 (1.02, 4.55)** | 1.84 (0.87, 3.90) | 1.81 (0.85, 3.82) |

Model 1 was adjusted for age, sex, and ethnicity.

Model 2 was adjusted for age, sex, ethnicity, household income, TDI, education level, employment status, smoking status, alcohol consumption, physical activity, and obesity.

Model 3 was adjusted for age, sex, ethnicity, household income, TDI, education level, employment status, smoking status, alcohol consumption, physical activity, obesity, history of hypertension, history of hyperlipidemia, and the PRS for T2DM.

Significant P values are in bold.

Abbreviations: CI, confidence interval; HR, hazard ratio; TDI, Townsend deprivation index; PRS, polygenic risk score.

**Supplementary Table S6. Associations Between Sleep Pattern Changes from Baseline to Follow-Up and the Risk of T2DM After Excluding Events Occurring Within the First Two Years (N=59,910).**

| **Sleep pattern changes** | **Cases/N** | **HR (95% CI)** | | |
| --- | --- | --- | --- | --- |
|  |  | **Model 1** | **Model 2** | **Model 3** |
| **All participants** | 945/59,910 | - | - | - |
| Stable poor | 18/351 | **3.93 (2.44, 6.34)** | **2.51 (1.55, 4.06)** | **2.35 (1.43, 3.85)** |
| Worsening | 202/11,620 | **1.78 (1.48, 2.13)** | **1.55 (1.29, 1.86)** | **1.53 (1.27, 1.85)** |
| Stable intermediate | 284/15,106 | **1.63 (1.38, 1.93)** | **1.30 (1.10, 1.54)** | **1.30 (1.09, 1.54)** |
| Optimizing | 178/8,681 | **1.77 (1.46, 2.14)** | **1.43 (1.18, 1.74)** | **1.36 (1.12, 1.66)** |
| Stable healthy | 263/24,152 | *Ref* | *Ref* | *Ref* |
| **Healthy sleep pattern at baseline** | 432/34,570 | *-* | - | - |
| Stable healthy | 263/24,152 | *Ref* | *Ref* | *Ref* |
| Worsening | 169/10,418 | **1.50 (1.24, 1.82)** | **1.36 (1.12, 1.65)** | **1.50 (1.23, 1.84)** |
| **Non-healthy sleep pattern at baseline^1^** | 513/25,340 | *-* | - | - |
| Non-worsening^2^ | 462/23,787 | *Ref* | *Ref* | *Ref* |
| Non-improvement^3^ | 51/1,553 | **1.92 (1.53, 2.42)** | **1.54 (1.22, 1.94)** | **1.37 (1.07, 1.74)** |

^1^ Non-healthy sleep pattern at baseline refers to a baseline sleep pattern that is intermediate or poor.

^2^ Non-worsening, including optimizing and stable intermediate from baseline.

^3^ Non-improvement, including worsening and stable poor from baseline.

Model 1 was adjusted for age, sex, and ethnicity.

Model 2 was adjusted for age, sex, ethnicity, household income, TDI, education level, employment status, smoking status, alcohol consumption, physical activity, and obesity.

Model 3 was adjusted for age, sex, ethnicity, household income, TDI, education level, employment status, smoking status, alcohol consumption, physical activity, obesity, history of hypertension, history of hyperlipidemia, and the PRS for T2DM.

Significant P values are in bold.

Abbreviations: CI, confidence interval; HR, hazard ratio; TDI, Townsend deprivation index; PRS, polygenic risk score.

**Supplementary Table S7. Person-Years, Incidence Rates, and Model-Standardized Four-Year Risk of Incident T2DM by Sleep Pattern Transition.**

| **Transition** | **Cases/N** | **Person-years** | **Incidence/1,000 PY (95% CI)** | **Unadjusted 4-y risk, %** | **Standardized 4-y risk, % (95% CI)** | **Risk difference vs stable healthy, %**  **(95% CI)*** |
| --- | --- | --- | --- | --- | --- | --- |
| Stable healthy | 357/24,246 | 98,616 | 3.62 (3.25, 4.02) | 0.99 | 1.06 (0.94, 1.20) | 0.00 (0.00, 0.00) |
| Stable poor | 21/354 | 1,485 | 14.14 (8.76, 21.62) | 2.56 | 2.13 (1.29, 2.93) | 1.07 (0.22, 1.87) |
| Worsening | 262/11,680 | 44,727 | 5.86 (5.17, 6.61) | 1.36 | 1.49 (1.28, 1.71) | 0.43 (0.20, 0.67) |
| Stable intermediate | 394/15,216 | 61,731 | 6.38 (5.77, 7.04) | 1.55 | 1.37 (1.25, 1.52) | 0.31 (0.12, 0.47) |
| Optimizing | 250/8,753 | 35,233 | 7.10 (6.24, 8.03) | 1.82 | 1.50 (1.26, 1.69) | 0.44 (0.22, 0.66) |

*Risk difference vs stable healthy.

Incidence-rate CIs are exact Poisson intervals. Unadjusted four-year risks were estimated as 1 minus the Kaplan–Meier survival probability at 4 years and therefore differ from crude event proportions over the full follow-up. Four-year adjusted risks are direct-standardized estimates from Model 3 pooled across 20 imputed datasets (N=60,249; 1,284 events). CIs for standardized risks and risk differences are percentile bootstrap intervals based on 200 successful resamples (seed 20260721).

Abbreviations: CI, confidence interval; PY, person-years; T2DM, type 2 diabetes mellitus.

**Supplementary Table S8. Association Between Signed Change in Healthy Sleep Score and Subsequent Risk of T2DM.**

| **Changes in HSS** | **HR (95% CI)** | | |
| --- | --- | --- | --- |
|  | **Model 1** | **Model 2** | **Model 3** |
| **No change** | Ref | Ref | Ref |
| **Decrease** | **1.17 (1.03, 1.34)** | **1.14 (1.00, 1.30)** | **1.14 (1.00, 1.30)** |
| **Increase** | **1.26 (1.10, 1.44)** | **1.16 (1.02, 1.33)** | **1.14 (1.00, 1.31)** |
| **Per 1 score increase** |  |  |  |
| All participants | 1.02 (0.97, 1.08) | 1.00 (0.95, 1.05) | 0.99 (0.94, 1.05) |
| HSS of 4–5 | **0.82 (0.75, 0.89)** | **0.85 (0.78, 0.93)** | **0.83 (0.76, 0.91)** |
| HSS of 0–3 | 1.02 (0.94, 1.10) | 1.03 (0.96, 1.11) | 1.05 (0.97, 1.13) |

Model 1 was adjusted for age, sex, and ethnicity.

Model 2 was adjusted for age, sex, ethnicity, household income, TDI, education level, employment status, smoking status, alcohol consumption, physical activity, and obesity.

Model 3 was adjusted for age, sex, ethnicity, household income, TDI, education level, employment status, smoking status, alcohol consumption, physical activity, obesity, history of hypertension, history of hyperlipidemia, and the PRS for T2DM.

Significant P values are in bold.

Abbreviations: CI, confidence interval; HR, hazard ratio; HSS, healthy sleep score; TDI, Townsend deprivation index; PRS, polygenic risk score.

**Supplementary Table S9. Participant and Event Counts Across Signed Changes in the HSS.**

| **Signed HSS change** | **N** | **Events** |
| --- | --- | --- |
| **Entire cohort** |  |  |
| -5 | 1 | 0 |
| -4 | 36 | 1 |
| -3 | 641 | 15 |
| -2 | 4,121 | 104 |
| -1 | 14,363 | 278 |
| 0 | 25,947 | 510 |
| 1 | 12,024 | 274 |
| 2 | 2,734 | 87 |
| 3 | 351 | 12 |
| 4 | 30 | 3 |
| 5 | 1 | 0 |
| **Baseline HSS 4** |  |  |
| -4 | 8 | 0 |
| -3 | 242 | 6 |
| -2 | 1,858 | 44 |
| -1 | 6,262 | 124 |
| 0 | 10,482 | 179 |
| 1 | 3,917 | 64 |
| **Baseline HSS 5** |  |  |
| -5 | 1 | 0 |
| -4 | 28 | 1 |
| -3 | 384 | 9 |
| -2 | 1,686 | 36 |
| -1 | 4,339 | 57 |
| 0 | 5,508 | 57 |

Signed change was defined as visit-2 HSS minus visit-1 HSS. Baseline HSS 0–3 comprised the remainder (N=25,534; events=707).

Abbreviations: HSS, healthy sleep score; T2DM, type 2 diabetes mellitus.

**Supplementary Table S10. Association Between Signed Change in Healthy Sleep Score and Subsequent Risk of T2DM After Excluding Events Occurring Within the First Two Years (N=59,910).**

| **Changes in HSS** | **HR (95% CI)** | | |
| --- | --- | --- | --- |
|  | **Model 1** | **Model 2** | **Model 3** |
| **No change** | Ref | Ref | Ref |
| **Decrease** | **1.24 (1.07, 1.45)** | **1.22 (1.05, 1.42)** | **1.22 (1.04, 1.42)** |
| **Increase** | **1.25 (1.07, 1.46)** | 1.16 (1.00, 1.36) | 1.10 (0.95, 1.31) |
| **Per 1 score increase** |  |  |  |
| All participants | 0.99 (0.92, 1.05) | 0.96 (0.91, 1.03) | 0.95 (0.89, 1.02) |
| HSS of 4–5 | **0.79 (0.71, 0.88)** | **0.82 (0.74, 0.91)** | **0.81 (0.73, 0.90)** |
| HSS of 0–3 | 0.99 (0.90, 1.08) | 1.00 (0.91, 1.09) | 1.02 (0.92, 1.10) |

Model 1 was adjusted for age, sex, and ethnicity.

Model 2 was adjusted for age, sex, ethnicity, household income, TDI, education level, employment status, smoking status, alcohol consumption, physical activity, and obesity.

Model 3 was adjusted for age, sex, ethnicity, household income, TDI, education level, employment status, smoking status, alcohol consumption, physical activity, obesity, history of hypertension, history of hyperlipidemia, and the PRS for T2DM.

Significant P values are in bold.

Abbreviations: CI, confidence interval; HR, hazard ratio; HSS, healthy sleep score; T2DM, type 2 diabetes mellitus; TDI, Townsend deprivation index; PRS, polygenic risk score.

**Supplementary Table S11. Association Between the Transition Patterns of Individual Sleep Dimensions and Subsequent Risk of T2DM.**

| **Changes in sleep patterns** | **HR (95% CI)** | | |
| --- | --- | --- | --- |
|  | **Model 1** | **Model 2** | **Model 3** |
| **Sleep duration change** |  |  |  |
| Stable Unhealthy | **1.67 (1.45, 1.93)** | **1.36 (1.18, 1.57)** | **1.35 (1.16, 1.56)** |
| Optimal change to non-optimal | **1.59 (1.37, 1.85)** | **1.41 (1.22, 1.64)** | **1.41 (1.21, 1.64)** |
| non-optimal change to optimal | **1.40 (1.18, 1.67)** | **1.21 (1.02, 1.44)** | 1.19 (0.99, 1.42) |
| **Chronotype change** |  |  |  |
| Stable Unhealthy | 1.03 (0.91, 1.17) | 0.97 (0.85, 1.10) | 0.97 (0.86, 1.11) |
| Morning change to evening | **1.41 (1.16, 1.73)** | **1.30 (1.06, 1.58)** | **1.31 (1.07, 1.61)** |
| Evening change to morning | **1.31 (1.10, 1.55)** | **1.19 (1.00, 1.42)** | **1.22 (1.02, 1.45)** |
| **Insomnia change** |  |  |  |
| Stable Unhealthy | **1.40 (1.21, 1.62)** | **1.27 (1.09, 1.47)** | **1.24 (1.07, 1.44)** |
| No insomnia change to insomnia | **1.45 (1.25, 1.68)** | **1.39 (1.20, 1.62)** | **1.39 (1.19, 1.62)** |
| Insomnia change to no insomnia | **1.30 (1.07, 1.58)** | 1.21 (0.99, 1.47) | 1.18 (0.97, 1.44) |
| **Snoring change** |  |  |  |
| Stable Unhealthy | **1.73 (1.52, 1.97)** | **1.31 (1.14, 1.49)** | **1.24 (1.08, 1.42)** |
| No snoring change to snoring | 1.03 (0.84, 1.26) | 0.91 (0.74, 1.12) | 0.92 (0.75, 1.13) |
| Snoring change to no snoring | **1.95 (1.66, 2.30)** | **1.49 (1.27, 1.76)** | **1.41 (1.19, 1.66)** |
| **Daytime dozing change** |  |  |  |
| Stable Unhealthy | **2.10 (1.32, 3.35)** | **2.05 (1.28, 3.27)** | **2.06 (1.27, 3.33)** |
| No dozing change to daytime dozing | **1.77 (1.31, 2.39)** | **1.45 (1.07, 1.96)** | **1.38 (1.01, 1.88)** |
| Daytime dozing change to no dozing | **1.55 (1.09, 2.20)** | 1.32 (0.93, 1.88) | 1.33 (0.93, 1.90) |

Model 1 was adjusted for age, sex, and ethnicity.

Model 2 was adjusted for age, sex, ethnicity, household income, TDI, education level, employment status, smoking status, alcohol consumption, physical activity, and obesity.

Model 3 was adjusted for age, sex, ethnicity, household income, TDI, education level, employment status, smoking status, alcohol consumption, physical activity, obesity, history of hypertension, history of hyperlipidemia, and the PRS for T2DM.

Stable healthy was the reference category.

Significant P values are in bold.

Abbreviations: CI, confidence interval; HR, hazard ratio; PRS, polygenic risk score; TDI, Townsend deprivation index; T2DM, type 2 diabetes mellitus.

**Supplementary Table S12. Subgroup Interaction Tests for the Ordered Sleep-Transition Code.**

| **Subgroup** | **Level** | **N** | **Events** | **Interaction P** | **BH q value** |
| --- | --- | --- | --- | --- | --- |
| Sex | Male | 28,298 | 782 | 0.493 | 0.619 |
|  | Female | 31,951 | 502 | 0.493 | 0.619 |
| Age | ≥65 | 5,897 | 173 | 0.005 | 0.044 |
|  | <65 | 54,352 | 1,111 | 0.005 | 0.044 |
| Ethnicity | White | 58,681 | 1,205 | 0.280 | 0.619 |
|  | Other | 1,568 | 79 | 0.280 | 0.619 |
| BMI | <25 kg/m² | 24,232 | 127 | 0.449 | 0.619 |
|  | 25–29.9 kg/m² | 25,694 | 523 | 0.449 | 0.619 |
|  | ≥30 kg/m² | 10,323 | 634 | 0.449 | 0.619 |
| Current smoking | Yes | 3,832 | 123 | 0.550 | 0.619 |
|  | No | 56,417 | 1,161 | 0.550 | 0.619 |
| Current drinking | No | 771 | 26 | 0.796 | 0.796 |
|  | Yes | 49,300 | 943 | 0.796 | 0.796 |
|  | Unknown | 10,178 | 315 | 0.796 | 0.796 |
| Hypertension | Yes | 12,179 | 536 | 0.127 | 0.381 |
|  | No | 48,070 | 748 | 0.127 | 0.381 |
| Hyperlipidemia | Yes | 4,273 | 208 | 0.039 | 0.174 |
|  | No | 55,976 | 1,076 | 0.039 | 0.174 |
| PRS for T2DM | Low | 20,083 | 180 | 0.544 | 0.619 |
|  | Intermediate | 20,083 | 373 | 0.544 | 0.619 |
|  | High | 20,083 | 731 | 0.544 | 0.619 |

HRs represent a one-category increase in the ordered transition code (1=stable poor, 2=worsening, 3=stable intermediate, 4=optimizing, 5=stable healthy).

The incompletely observed PRS was multiply imputed in 20 datasets. Interaction tests were pooled across imputations, and q values were adjusted using Benjamini–Hochberg correction across nine tests.

Abbreviations: BH, Benjamini–Hochberg; BMI, body mass index; PRS, polygenic risk score; T2DM, type 2 diabetes mellitus.

**Supplementary Table S13. Sensitivity Analysis of Sleep Pattern Transitions and Incident T2DM Using Complete Cases (N=58,544).**

| **Sleep pattern changes** | **Cases/N** | **HR (95% CI)** | | |
| --- | --- | --- | --- | --- |
|  |  | **Model 1** | **Model 2** | **Model 3** |
| **All participants** | 1,234/58,544 | - | - | - |
| Stable poor | 20/337 | **3.48 (2.22, 5.47)** | **2.13 (1.36, 3.36)** | **2.05 (1.30, 3.23)** |
| Worsening | 252/11,359 | **1.66 (1.41, 1.95)** | **1.42 (1.20, 1.67)** | **1.42 (1.20, 1.67)** |
| Stable intermediate | 381/14,768 | **1.69 (1.46, 1.96)** | **1.32 (1.14, 1.53)** | **1.30 (1.12, 1.51)** |
| Optimizing | 237/8,419 | **1.83 (1.55, 2.16)** | **1.46 (1.23, 1.72)** | **1.41 (1.19, 1.67)** |
| Stable healthy | 344/23,661 | *Ref* | *Ref* | *Ref* |
| **Healthy sleep pattern at baseline** |  | *-* | - | - |
| Stable healthy | 344/23,661 | *Ref* | *Ref* | *Ref* |
| Worsening | 212/10,182 | **1.57 (1.32, 1.86)** | **1.39 (1.17, 1.65)** | **1.41 (1.19, 1.68)** |
| **Non-healthy sleep pattern at baseline^1^** |  | *-* | - | - |
| Non-worsening^2^ | 618/23,187 | *Ref* | *Ref* | *Ref* |
| Non-improvement^3^ | 60/1,514 | **1.51 (1.16, 1.97)** | **1.26 (1.07, 1.49)** | **1.20 (1.01, 1.43)** |

^1^ Non-healthy sleep pattern at baseline refers to a baseline sleep pattern that is intermediate or poor.

^2^ Non-worsening, including optimizing and stable intermediate from baseline.

^3^ Non-improvement, including worsening and stable poor from baseline.

Model 1 was adjusted for age, sex, and ethnicity.

Model 2 was adjusted for age, sex, ethnicity, household income, TDI, education level, employment status, smoking status, alcohol consumption, physical activity, and obesity.

Model 3 was adjusted for age, sex, ethnicity, household income, TDI, education level, employment status, smoking status, alcohol consumption, physical activity, obesity, history of hypertension, history of hyperlipidemia, and the PRS for T2DM.

Significant P values are in bold.

Abbreviations: CI, confidence interval; HR, hazard ratio; PRS, polygenic risk score; T2DM, type 2 diabetes mellitus; TDI, Townsend deprivation index.

**Supplementary Table S14. Sensitivity Analyses Using Alternative Linear and Nonlinear BMI Specifications.**

| **Sleep Pattern Transition** | **Cases/N** | **HR (95% CI)** |
| --- | --- | --- |
|  |  | **Model 3** |
| **Continuous BMI** | **1,284/60,249** | **-** |
| Stable poor | 21/354 | **1.99 (1.28, 3.11)** |
| Worsening | 262/11,680 | **1.39 (1.18, 1.63)** |
| Stable intermediate | 394/15,216 | **1.23 (1.06, 1.42)** |
| Optimizing | 250/8,753 | **1.34 (1.13, 1.57)** |
| Stable healthy | 357/24,246 | Ref |
| **Nonlinear BMI** | **1,284/60,249** | **-** |
| Stable poor | 21/354 | **1.84 (1.18, 2.87)** |
| Worsening | 262/11,680 | **1.36 (1.16, 1.60)** |
| Stable intermediate | 394/15,216 | **1.20 (1.04, 1.39)** |
| Optimizing | 250/8,753 | **1.33 (1.13, 1.56)** |
| Stable healthy | 357/24,246 | Ref |

Continuous BMI replaced the obesity indicator in Model 3; nonlinear BMI was modeled using a natural cubic spline with 3 degrees of freedom. The pooled joint test for the nonlinear terms gave P < 0.001.

Model 3 was adjusted for age, sex, ethnicity, household income, TDI, education level, employment status, smoking status, alcohol consumption, physical activity, history of hypertension, history of hyperlipidemia, and the PRS for T2DM.

Statistically significant estimates are in bold.

Abbreviations: BMI, body mass index; CI, confidence interval; HR, hazard ratio; PRS, polygenic risk score; T2DM, type 2 diabetes mellitus; TDI, Townsend deprivation index.

**Supplementary Table S15. Cause-Specific Competing-Risk Analysis Accounting for Death.**

| **Sleep pattern changes** | **Cases/N** | **HR (95% CI)** | | |
| --- | --- | --- | --- | --- |
|  |  | **Model 1** | **Model 2** | **Model 3** |
| **All participants** | 1,284/60,249 | - | - | - |
| Stable poor | 21/354 | **3.41 (2.19, 5.30)** | **2.11 (1.35, 3.29)** | **2.03 (1.30, 3.16)** |
| Worsening | 262/11,680 | **1.66 (1.42, 1.95)** | **1.42 (1.21, 1.67)** | **1.42 (1.21, 1.66)** |
| Stable intermediate | 394/15,216 | **1.68 (1.45, 1.94)** | **1.32 (1.14, 1.53)** | **1.30 (1.13, 1.51)** |
| Optimizing | 250/8,753 | **1.84 (1.57, 2.17)** | **1.47 (1.25, 1.73)** | **1.43 (1.21, 1.68)** |
| Stable healthy | 357/24,246 | *Ref* | *Ref* | *Ref* |
| **Healthy sleep pattern at baseline** |  | *-* | - | - |
| Stable healthy | 357/24,246 | *Ref* | *Ref* | *Ref* |
| Worsening | 220/10,469 | **1.57 (1.33, 1.86)** | **1.39 (1.18, 1.65)** | **1.41 (1.19, 1.67)** |
| **Non-healthy sleep pattern at baseline^1^** |  | *-* | - | - |
| Non-worsening^2^ | 644/23,969 | *Ref* | *Ref* | *Ref* |
| Non-improvement^3^ | 63/1,565 | **1.51 (1.17, 1.96)** | **1.33 (1.09, 1.62)** | **1.21 (1.00, 1.46)** |

^1^ Non-healthy sleep pattern at baseline refers to a baseline sleep pattern that is intermediate or poor.

^2^ Non-worsening, including optimizing and stable intermediate from baseline.

^3^ Non-improvement, including worsening and stable poor from baseline.

Model 1 was adjusted for age, sex, and ethnicity.

Model 2 was adjusted for age, sex, ethnicity, household income, TDI, education level, employment status, smoking status, alcohol consumption, physical activity, and obesity.

Model 3 was adjusted for age, sex, ethnicity, household income, TDI, education level, employment status, smoking status, alcohol consumption, physical activity, obesity, history of hypertension, history of hyperlipidemia, and the PRS for T2DM.

The cause-specific Cox model censored participants at death and estimated cause-specific hazards. One record whose recorded death date preceded Visit 2 was retained and censored at its existing positive 4-day post-landmark follow-up time; no death time was imputed.

Significant P values are in bold.

Abbreviations: CI, confidence interval; HR, hazard ratio; PRS, polygenic risk score; T2DM, type 2 diabetes mellitus; TDI, Townsend deprivation index.

**Supplementary Table S16. Fine–Gray Competing-Risk Analysis Accounting for Death.**

| **Sleep pattern changes** | **Cases/N** | **HR (95% CI)** | | |
| --- | --- | --- | --- | --- |
|  |  | **Model 1** | **Model 2** | **Model 3** |
| **All participants** | 1,284/60,249 | - | - | - |
| Stable poor | 21/354 | **3.44 (2.20, 5.38)** | **2.13 (1.36, 3.33)** | **2.05 (1.33, 3.15)** |
| Worsening | 262/11,680 | **1.66 (1.41, 1.95)** | **1.42 (1.21, 1.67)** | **1.42 (1.21, 1.67)** |
| Stable intermediate | 394/15,216 | **1.68 (1.45, 1.94)** | **1.32 (1.14, 1.53)** | **1.30 (1.12, 1.51)** |
| Optimizing | 250/8,753 | **1.85 (1.57, 2.17)** | **1.48 (1.25, 1.74)** | **1.43 (1.21, 1.68)** |
| Stable healthy | 357/24,246 | *Ref* | *Ref* | *Ref* |
| **Healthy sleep pattern at baseline** |  | *-* | - | - |
| Stable healthy | 357/24,246 | *Ref* | *Ref* | *Ref* |
| Worsening | 220/10,469 | **1.57 (1.32, 1.86)** | **1.39 (1.17, 1.66)** | **1.41 (1.19, 1.68)** |
| **Non-healthy sleep pattern at baseline^1^** |  | *-* | - | - |
| Non-worsening^2^ | 644/23,969 | *Ref* | *Ref* | *Ref* |
| Non-improvement^3^ | 63/1,565 | **1.52 (1.17, 1.97)** | **1.33 (1.10, 1.62)** | **1.22 (1.02, 1.46)** |

^1^ Non-healthy sleep pattern at baseline refers to a baseline sleep pattern that is intermediate or poor.

^2^ Non-worsening, including optimizing and stable intermediate from baseline.

^3^ Non-improvement, including worsening and stable poor from baseline.

Model 1 was adjusted for age, sex, and ethnicity.

Model 2 was adjusted for age, sex, ethnicity, household income, TDI, education level, employment status, smoking status, alcohol consumption, physical activity, and obesity.

Model 3 was adjusted for age, sex, ethnicity, household income, TDI, education level, employment status, smoking status, alcohol consumption, physical activity, obesity, history of hypertension, history of hyperlipidemia, and the PRS for T2DM.

Subdistribution HRs were estimated using the Fine–Gray method with survival::finegray.

Statistically significant estimates are in bold.

Abbreviations: CI, confidence interval; HR, hazard ratio; PRS, polygenic risk score; T2DM, type 2 diabetes mellitus; TDI, Townsend deprivation index.

**Supplementary Table S17. Sensitivity Analysis Excluding Participants with Baseline Prediabetes (N=27,864).**

| **Sleep pattern changes** | **Cases/N** | **HR (95% CI)** | | |
| --- | --- | --- | --- | --- |
|  |  | **Model 1** | **Model 2** | **Model 3** |
| **All participants** | 288/27,864 | - | - | - |
| Stable poor | 6/154 | **5.20 (2.26, 11.96)** | **2.69 (1.16, 6.24)** | **2.42 (1.04, 5.66)** |
| Worsening | 58/5,563 | **1.75 (1.24, 2.47)** | **1.47 (1.04, 2.08)** | **1.47 (1.04, 2.08)** |
| Stable intermediate | 94/6,806 | **2.03 (1.50, 2.76)** | **1.54 (1.13, 2.10)** | **1.52 (1.11, 2.06)** |
| Optimizing | 55/3,831 | **2.04 (1.44, 2.90)** | **1.64 (1.15, 2.32)** | **1.59 (1.12, 2.26)** |
| Stable healthy | 75/11,510 | *Ref* | *Ref* | *Ref* |
| **Healthy sleep pattern at baseline** |  | *-* | - | - |
| Stable healthy | 75/11,510 | *Ref* | *Ref* | *Ref* |
| Worsening | 49/4,997 | **1.66 (1.16, 2.38)** | **1.45 (1.01, 2.09)** | **1.43 (0.99, 2.06)** |
| **Non-healthy sleep pattern at baseline^1^** |  | *-* | - | - |
| Non-worsening^2^ | 149/10,637 | *Ref* | *Ref* | *Ref* |
| Non-improvement^3^ | 15/720 | 1.52 (0.89, 2.59) | 1.22 (0.71, 2.08) | 1.18 (0.69, 2.03) |

^1^ Non-healthy sleep pattern at baseline refers to a baseline sleep pattern that is intermediate or poor.

^2^ Non-worsening, including optimizing and stable intermediate from baseline.

^3^ Non-improvement, including worsening and stable poor from baseline.

Model 1 was adjusted for age, sex, and ethnicity.

Model 2 was adjusted for age, sex, ethnicity, household income, TDI, education level, employment status, smoking status, alcohol consumption, physical activity, and obesity.

Model 3 was adjusted for age, sex, ethnicity, household income, TDI, education level, employment status, smoking status, alcohol consumption, physical activity, obesity, history of hypertension, history of hyperlipidemia, and the PRS for T2DM.

Prediabetes was defined according to American Diabetes Association criteria as fasting plasma glucose 5.6–6.9 mmol/L or glycated hemoglobin (HbA1c) 5.7%–6.4% (39–46 mmol/mol).

Visit-2 prediabetes could not be assessed because glucose and HbA1c were unavailable at Instance 2.

Significant P values are in bold.

Abbreviations: CI, confidence interval; HbA1c, glycated hemoglobin; HR, hazard ratio; PRS, polygenic risk score; T2DM, type 2 diabetes mellitus; TDI, Townsend deprivation index.

**Supplementary Table S18. Schoenfeld Residual Tests for Model 3.**

| **Term** | **Chi-square** | **Degrees of freedom** | **P value** |
| --- | --- | --- | --- |
| Sleep pattern transition | 9.98 | 4 | 0.041 |
| Age | 1.02 | 1 | 0.314 |
| Sex | 1.42 | 1 | 0.233 |
| Ethnicity | 2.81 | 1 | 0.093 |
| Household income | 7.48 | 3 | 0.058 |
| Townsend deprivation index | 0.02 | 1 | 0.885 |
| University degree | 5.02 | 2 | 0.081 |
| Employment status | 0.30 | 1 | 0.581 |
| Smoking status | 2.65 | 2 | 0.265 |
| Alcohol consumption status | 0.64 | 3 | 0.887 |
| Physical activity | 1.87 | 3 | 0.599 |
| Obesity | 0.00 | 1 | 0.955 |
| Hypertension | 1.65 | 1 | 0.199 |
| Hyperlipidemia | 2.10 | 1 | 0.147 |
| Polygenic risk score for T2DM | 0.23 | 1 | 0.630 |
| Global test | 34.33 | 26 | 0.127 |

Schoenfeld residual tests were based on Model 3. The global test was P = 0.127; the four-degree-of-freedom sleep-transition term test was P = 0.041.

Abbreviations: T2DM, type 2 diabetes mellitus.

**Supplementary Table S19. Multiple-Imputation Sensitivity Analysis Including All Incomplete Covariates.**

| **Changes in HSS** | **HR (95% CI)** | | |
| --- | --- | --- | --- |
|  | **Model 1** | **Model 2** | **Model 3** |
| **No change** | Ref | Ref | Ref |
| **Decrease** | 1.173 (1.029, 1.337) | 1.140 (1.000, 1.300) | 1.151 (1.010, 1.313) |
| **Increase** | 1.262 (1.104, 1.442) | 1.159 (1.014, 1.324) | 1.149 (1.005, 1.314) |
| **Per 1 score increase** |  |  |  |
| All participants | 1.024 (0.969, 1.081) | 0.999 (0.947, 1.054) | 0.994 (0.941, 1.049) |
| HSS of 4–5 | 0.817 (0.746, 0.893) | 0.851 (0.778, 0.930) | 0.840 (0.768, 0.919) |
| HSS of 0–3 | 1.019 (0.944, 1.099) | 1.030 (0.956, 1.110) | 1.045 (0.969, 1.126) |

Model 1 was adjusted for age, sex, and ethnicity.

Model 2 was adjusted for age, sex, ethnicity, household income, TDI, education level, employment status, smoking status, alcohol consumption, physical activity, and obesity.

Model 3 was adjusted for age, sex, ethnicity, household income, TDI, education level, employment status, smoking status, alcohol consumption, physical activity, obesity, history of hypertension, history of hyperlipidemia, and the PRS for T2DM.

All covariates treated as incomplete in this sensitivity analysis, including university degree, household income, alcohol consumption, and the PRS, were multiply imputed.

Twenty imputed datasets were generated by fully conditional specification. Logistic or multinomial regression was used for categorical variables, predictive mean matching for PRS, and the T2DM indicator and Nelson–Aalen cumulative hazard were included as predictors; estimates were pooled using Rubin’s rules.

Abbreviations: CI, confidence interval; HR, hazard ratio; PRS, polygenic risk score; T2DM, type 2 diabetes mellitus; TDI, Townsend deprivation index.

**Supplementary Table S20. Associations of Sleep Pattern Transitions with the Visit-1 TyG Index (N=51,450).**

| **Sleep patterns** | **β (95% CI)** | **P value** |
| --- | --- | --- |
| **All participants** |  |  |
| Stable poor | 0.097 (0.040, 0.154) | <0.001 |
| Worsening | 0.020 (0.008, 0.032) | <0.001 |
| Stable intermediate | 0.053 (0.042, 0.064) | <0.001 |
| Optimizing | 0.035 (0.022, 0.049) | <0.001 |
| Stable healthy reference | *Ref* | *Ref* |
| **Baseline poor** |  |  |
| Intermediate | 0.015 (-0.053, 0.084) | 0.659 |
| Healthy | -0.006 (-0.114, 0.102) | 0.908 |
| Poor | *Ref* | *Ref* |
| **Baseline intermediate** |  |  |
| Poor | 0.019 (-0.013, 0.051) | 0.246 |
| Healthy | -0.026 (-0.041, -0.011) | <0.001 |
| Intermediate | *Ref* | *Ref* |
| **Baseline healthy** |  |  |
| Intermediate | 0.015 (0.003, 0.027) | 0.016 |
| Poor | 0.001 (-0.062, 0.064) | 0.975 |
| Healthy | Ref | Ref |

TyG was calculated as ln[triglyceride (mg/dL) × glucose (mg/dL)/2] using visit-1 laboratory measurements.

Model was adjusted for age, sex, ethnicity, household income, TDI, education level, employment status, smoking status, alcohol consumption, physical activity, obesity, history of hypertension, history of hyperlipidemia, and the PRS for T2DM.

Abbreviations: CI, confidence interval; T2DM, type 2 diabetes mellitus; TyG, triglyceride-glucose index.

**Figure Legends**

**Supplementary Figure S1. Study flow chart**

Abbreviations: HbA1c, glycated hemoglobin; T1DM, type 1 diabetes mellitus; T2DM, type 2 diabetes mellitus; UKB, UK Biobank. Among the 5,935 participants excluded for baseline diabetes, 372 had T1DM and 5,563 had T2DM.

**Supplementary Figure S2. Directed acyclic graph for the prespecified covariate-adjustment framework.**

Gray arrows indicate hypothesized common-cause relations between demographic and genetic, socioeconomic, and lifestyle and clinical factors and both sleep-pattern transitions and incident T2DM. The blue arrow denotes the exposure–outcome association. Gold arrows indicate that visit-1 TyG and pre-transition metabolic state precede the sleep transition; TyG was treated as an exploratory marker rather than a mediator. Dashed arrows denote conditioning at the visit-2 landmark, when participants were alive and diabetes-free.

Abbreviations: BMI, body mass index; PRS, polygenic risk score; T2DM, type 2 diabetes mellitus; TDI, Townsend deprivation index; TyG, triglyceride-glucose index.

**Supplementary Figure S3. Nonlinear dose-response relationship between signed change in the healthy sleep score and incident T2DM by baseline sleep pattern.** (A) Participants with HSS of 0–3; (B) Participants with HSS of 4–5.

Panel A used knots at signed HSS changes of −1, 0, and 2 (N=25,534; 707 events), and panel B used knots at −2, 0, and 1 (N=34,715; 577 events); 0 was the reference in both panels. The overall and nonlinearity P values were 0.074 and 0.036 for panel A and <0.001 and 0.048 for panel B, respectively. The associations were modeled using restricted cubic splines. The shaded areas represent the 95% CIs. Models were adjusted for age, sex, ethnicity, household income, TDI, education level, employment status, smoking status, alcohol consumption, physical activity, obesity, history of hypertension, history of hyperlipidemia, and the PRS for T2DM.

Abbreviations: CI, confidence interval; HR, hazard ratio; HSS, healthy sleep score; TDI, Townsend deprivation index; PRS, polygenic risk score.

**Supplementary Figure S4. Nonlinear dose-response relationship between signed change in the healthy sleep score and incident T2DM after excluding events occurring within the first two years.** (A) All participants; (B) Participants with HSS of 0–3; (C) Participants with HSS of 4; (D) Participants with HSS of 5.

Panels A–D used knots at signed HSS changes of −1, 0, and 1; −1, 0, and 2; −1, 0, and 1; and −2, −1, and 0, respectively, with 0 as the reference. The corresponding sample sizes/events were 59,910/945, 25,340/513, 22,664/312, and 11,906/120. The overall P values were <0.001, 0.153, 0.004, and <0.001, and the nonlinearity P values were <0.001, 0.059, <0.001, and 0.184, respectively. The associations were modeled using restricted cubic splines. The shaded areas represent the 95% CIs. Models were adjusted for age, sex, ethnicity, household income, TDI, education level, employment status, smoking status, alcohol consumption, physical activity, obesity, history of hypertension, history of hyperlipidemia, and the PRS for T2DM.

Abbreviations: CI, confidence interval; HR, hazard ratio; HSS, healthy sleep score; TDI, Townsend deprivation index; PRS, polygenic risk score.
